# Scoring the EQ-HWB-S: can we do it without value sets? A non-parametric item response theory analysis

**DOI:** 10.1007/s11136-024-03601-7

**Published:** 2024-02-21

**Authors:** You-Shan Feng, Thomas Kohlmann, Tessa Peasgood, Lidia Engel, Brendan Mulhern, A. Simon Pickard

**Affiliations:** 1 Institute for Clinical Epidemiology and Applied Biometrics, Medical University of Tübingen, Silcherstraße 5, 72076 Tübingen, Germany; 2 Institute for Community Medicine, University of Greifswald, Greifswald, Germany; 3 Division of Population Health, School of Medicine and Population Health, University of Sheffield, Sheffield, UK; 4 Monash University Health Economics Group, School of Public Health and Preventive Medicine, Monash University, Melbourne, VIC Australia; 5 Centre for Health Economics Research and Evaluation, University of Technology Sydney, Sydney, Australia; 6 Department of Pharmacy Systems, Outcomes and Policy, College of Pharmacy, University of Illinois Chicago, Chicago, IL USA

**Keywords:** Mokken scaling, EQ-HWB-S, Level sum score, Non-parametric item response theory, Unweighted summary score, Non-preference scoring approaches

## Abstract

**Background:**

Only one pilot value set (UK) is currently available for the EQ Health and Wellbeing Instrument short version (EQ-HWB-S). As an alternative to preference-weighted scoring, we examined whether a level summary score (LSS) is appropriate for the EQ-HWB-S using Mokken scaling analyses.

**Methods:**

Data from patients, carers and the general population collected during the developmental phase of the EQ-HWB-S in Australia, US and UK were used, noting 3 of 9 items have since undergone revision. EQ-HWB-S data fit was examined using R package Mokken scaling’s monotone homogeneity model, utilizing the automated item selection procedure (AISP) as well as Loevinger’s scaling coefficients for items and the scale (H_S_). Manifest monotonicity was assessed by examining whether the cumulative probability for responses at or above each response level did not decrease across the summary score.

**Results:**

EQ-HWB-S data were available for 3340 respondents: US = 903, Australia = 514 and UK = 1923. Mean age was 50 ± 18 and 1841 (55%) were female. AISP placed all 9 items of the EQ-HWB-S on a single scale when the lower bound was set to < 0.448. Strong scalability (H_S_ = 0.561) was found for the EQ-HWB-S as a single scale. Stronger scales were formed by separating the psychosocial items (*n* = 6, H_S_ = 0.683) and physical sensation items (*n* = 3, H_S_ = 0.713). No violations of monotonicity were found except for the items mobility and daily activities for the subgroups with long-term conditions and UK subjects, respectively.

**Discussion:**

As EQ-HWB-S items formed a strong scale and subscales based on Mokken analysis, LSS is a promising weighting-free approach to scoring.

**Supplementary Information:**

The online version contains supplementary material available at 10.1007/s11136-024-03601-7.

## Background

The EQ health and wellbeing (EQ-HWB) is a self-reported measure intended to inform resource allocation across health and social care settings [1–3]. Its development was motivated by the need to include domains relevant to well-being in the context of health and social care which may not be covered by existing health-related quality of life measures [2–5]. The instrument was developed using qualitative and quantitative methods and its short version, the EQ-HWB-S, comprises 9 items. Preference-based scoring, which involve eliciting values from country-specific populations [6], are under development for the EQ-HWB-S but currently, only a pilot preference value set (for UK [7]) is available and there is no non-preference based scoring.

A simple method to provide a summary score for the EQ suite of instruments is to sum the ordinal response levels of items [8]. This approach, sometimes called the “equally weighted” score [9], “unweighted” scoring approach [10, 11], or “level sum score” (LSS) [12], can be applied when preference-based scoring is not possible or available. The appeal of the LSS is its simplicity and that it is the same across countries and populations [13, 14]. Previous investigations revealed substantial agreement and similar psychometric properties between the LSS and utility weighted scores [9–11]. A recent paper found support for ordering respondents on the EQ-5D-5L using the LSS [8]. Whether the LSS is an appropriate method for scoring the EQ-HWB-S remains unclear.

Item response theory (IRT) is a family of models that assess the relationship between a latent (not directly observable) construct (theta *θ*) and the manifest (observable) response patterns of a set of items. The probability of endorsing a particular response level on items of a scale is dependent on the respondent’s level of *θ*. Non-parametric item response theory (NP-IRT) approaches do not make strict assumptions about the patterns of response probabilities [15]. Mokken scaling, the most well-known NP-IRT approach, does not estimate the exact level of *θ* but rather examines whether respondents can be ordered along the *θ*, and whether items can be ordered using the mean item scores given the response patterns of a dataset [15, 16]. If the LSS is a proxy for *θ*, then ordering of respondents along the LSS should reflect the ordering of persons along *θ*.

The aim of this study was to investigate if EQ-HWB-S data fits Mokken scaling models, which would support the use of the LSS. The LSS would allow for scoring of the EQ-HWB-S when no preference-based value set is available and also for comparisons across populations.

## Methods

### The EQ-HWB-S

The EQ-HWB-S is a short version of the 25-item EQ-HWB [17, 18] and comprises 9 items covering mobility, daily activities, coping (control), concentrating/thinking, anxiety, depression (sad), loneliness, fatigue, and pain. Response levels indicate frequency, level of difficulty, or severity: all use five-level ordinal response format covering the same recall period (the last 7 days), making the instruments ideal for LSS and Mokken scaling approaches [1, 4, 5]. In lieu of a set of commonly agreed upon labels for the items, we use abbreviations in this manuscript as described in Appendix B.

### EQ-HWB psychometric study

Secondary data was available: as part of the development of the EQ-HWB instruments, a large multi-country psychometric study was conducted to test more than 60 candidate items [3, 4]. We limited these analyses to the English language version of the EQ-HWB-S—data from Australia (AUS), United Kingdom (UK) and the United States of America (US)—in order to limit cross-linguistic differences which can affect measurement properties. The psychometric survey was conducted using both face-to-face and online methods, and described in detail in Peasgood et al. [3]. The general population, carer, and specific patient groups were recruited for each country: US cancer patients, UK patients with cancer, depression, diabetes, arthritis, heart conditions, irritable bowel syndrome/Crohn’s disease, and AUS patients with mental and physical health problems and/or those experiencing pain. Respondents completed a battery of health status, well-being, social care measures, alongside the EQ-HWB item pool.

Because no new data were collected for this study, no additional ethical approval was required beyond the approvals gained in each country for the data collection. Permission to share the data with research groups was given in the initial consent obtained during the psychometric study. Data management was handled in Microsoft Excel while statistical analyses were conducted using Stata SE 16 [19] and the statistical language and environment R [20]. Mokken analyses were conducted using van der Ark’s package “mokken” [21, 22] and the corresponding R script is available in Appendix A.

### Descriptive analysis

Simple descriptive analysis was conducted on socio-demographic and health-related variables avaliable in the dataset. We used mean and standard deviations to describe continuous, and count and percentages to describe categorical variables. Descriptive analysis was conducted for the complete data set as well as stratified by countries. The EQ-5D-5L was scored using both the LSS approach [8] and using the US value set [23].

### Item preparation

Some candidate items included in the EQ-HWB psychometric study were further refined after the study concluded. Therefore, several items did not match the exact wording of the current (2023) experimental version of the EQ-HWB-S (See Monteiro et al. Table 1, for current item wording [24]). Three “control” items were included in the psychometric study: the negative control item without examples exhibited the best performance and was selected for analysis. Three EQ-HWB-S items underwent substantive revisions and therefore no equivalent item was included in the psychometric study: (1) mobility inside/outside, (2) concentrating/thinking, and (3) feeling sad/depressed (the text of these items as worded for the psychometric study and their wording in the current version of the EQ-HWB-S can be found in Appendix C).

**Fig. 1:**
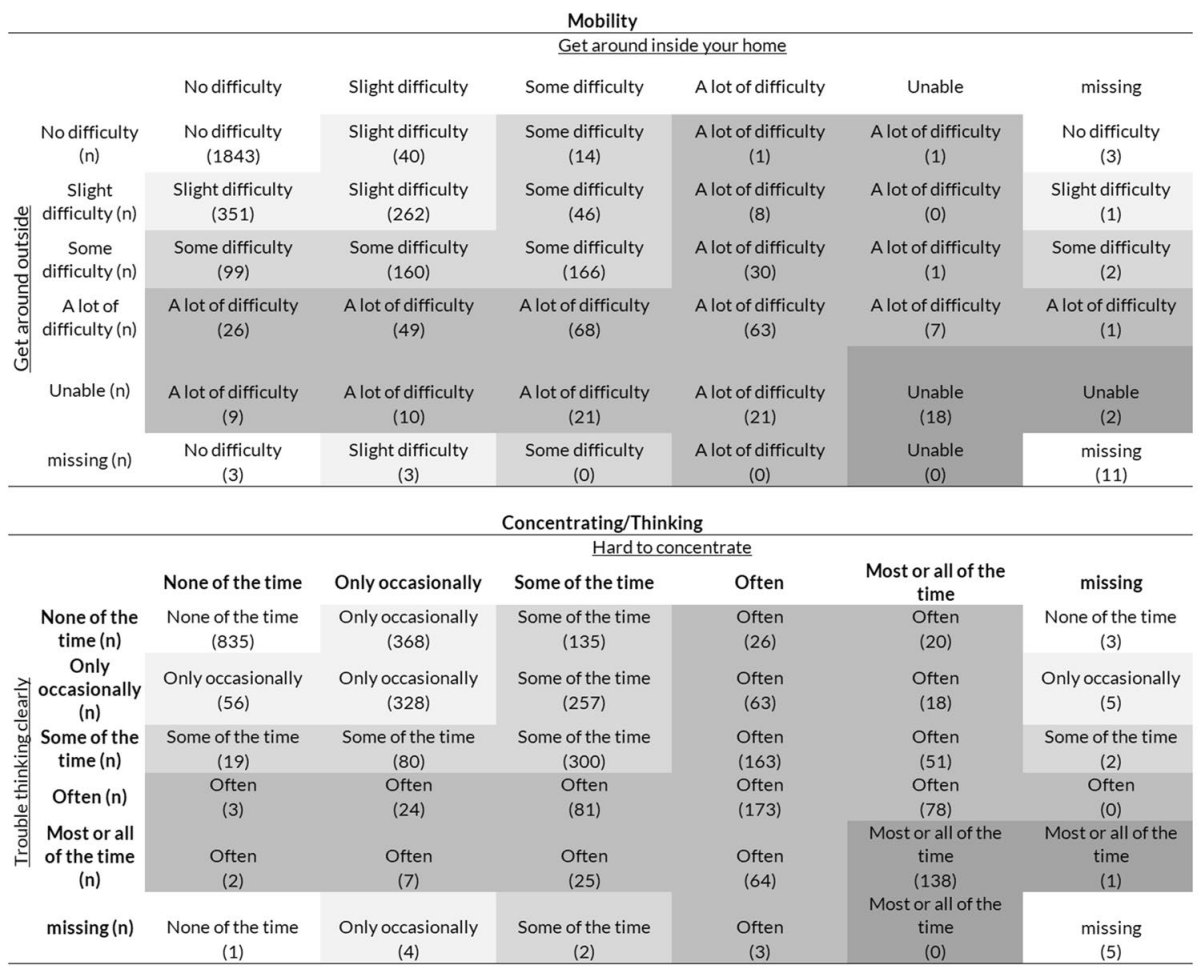
Combining “mobility” and “concentrating/thinking” items of the psychometric study

The psychometric study included items about difficulty “to get around” (1) inside and (2) outside, as well as items about problems with (3) concentrating and (4) thinking clearly. Using a similar strategy as earlier work that combined two items into a single dimension [25], we created the composite items “mobility” and “concentrating/thinking” by combining items (1) and (2), and items (3) and (4), respectively. Responses of the pairs of items were used to create new composite variables as detailed in Fig. 1. The four original items as well as the two composites were tested in models.

The UK and US psychometric studies contained an item asking about sadness but not depression: therefore a composite “feeling sad/depressed” could not be created. However, a “depression” item (using the same response format as the “sad” item) was included in the AUS study as it was found to have face validity in previous qualitative work. A subgroup analysis specifically addressing the composite “feeling sad/depressed” item was carried out using the AUS dataset.

### Mokken scale analysis

Mokken scaling is a set of non-parametric item response theory-based tools, consisting mainly of two nested models that can elucidate the ordinal location of respondents and items along a latent trait θ: the monotone homogeneity (MHM) and double monotonicity (DMM) models [26, 27]. Individuals are ordered according to the unweighted summary scores of their responses and items are ordered according to mean scores [15, 26, 27]. The polytomous MHM, which test model assumptions at the item and at the rating scale levels, was used [28, 29].

### The monotone homogeneity model (MHM) and scalability

The three assumptions of the MHM are:

1. Unidimensionality (items within the scale measure the same underlying latent trait);
2. Local independence (correlations between responses to scale items are influenced only by the level of *θ*); and
3. Monotonicity (the probability of endorsing particular response levels is monotonically non-decreasing as *θ* increases).

Loevinger’s homogeneity coefficients H were used to assess scalability of the EQ-HWB-S items. We examined H on the item (H_i_) and scale (H_S_) levels. Where the ‘rest score’ was the summary score minus the score of the item of interest, H_i_ was the normed covariance between item and rest scores while H_S_ was the weighted mean of H_i_ [15, 21]. The closer H_i_ is to 1, the better an item can discriminate subjects along *θ* within a scale. Negative H_i_ indicates a violation of MHM. The commonly accepted rules of thumb for H_S_ were used: H_S_ < 0.3 was unacceptable, H_S_ 0.3–0.4 indicated a weak scale, H_S_ 0.4–0.5 was interpreted as moderate and H_S_ ≥ 0.5 as strong ([30], pp. 60–61).

Automated item selection procedure (AISP) is a standard feature of the R package ‘mokken’ which identifies items that order persons well in a scale or scales [21]. Although the lower bound of H_i_ > 0.3 has been suggested for accepting items within a scale, Sijtsma and van der Ark suggested exploring different lower bound values smaller and larger than 0.3 [15, 21]. We executed AISP 12 times with the lower bound set between 0.1 and 0.6, increasing in steps of 0.05 (results are presented in steps of 0.1). The level of H_i_ at which one scale was no longer appropriate was identified by adjusting the lower bound using steps of 0.001. For this analysis, we made no assumptions about the structure of the EQ-HWB (i.e. if items belonged to different subscales or if all items can be scaled together).

Monotonicity is the property that as the level of the latent trait θ increases, the probability for endorsing at least a certain response level in an item does not decrease (i.e. the probability either remains stable or increases) [26, 27]. Latent monotonicity generally implies manifest monotonicity, which is observable in the data [21]. Manifest monotonicity was assessed by examining whether the cumulative probability for an item-level rating at or above each item-level rating was not decreasing as rest score increased (as defined by rest-score groups). Rest score groups were created automatically based on minimum sample size requirements for each group and only violations greater than the default minimum (*minvi* > 0.03) were reported [21, 22]. Item step response functions (ISRFs) plot the probability for endorsing a response level or higher across the latent variable and the item response function (IRF) for polytomous items is the sum of items’ ISRFs. IRFs were visually inspected for monotonicity.

### Invariant item ordering

The DMM model has the additional assumption that the IRF/ISRF of items do not intersect and is generally not meaningful for scales with polytomous items [31]. Instead of examining DMM, we assessed manifest invariant item ordering (MIIO) as suggested by Ligtvoet et al. [32, 33]. If items can be ordered along *θ*, then that order can facilitate interpretations for the EQ-HWB-S LSS [15, 21, 22, 31]. MIIO was implemented in the R package “mokken” using the check.iio function which ordered items by their conditional mean scores and checked each item pair for violations of ordering in rest score groups. Violations exceeding the default minimum value (number of ISRFs times 0.03) are reported [22, 33]. We also examined coefficient H^T^ which indicated the degree to which the sample data followed item ordering. The rules of thumb for H^T^ were used: H^T^ < 0.3 as items cannot be ordered, H^T^ 0.3 to 0.4 as low, H^T^ 0.4 to 0.5 as accurate and H^T^ > 0.5 as highly accurate [33].

### Exploratory factor analysis (EFA)

While not the focus of this study, EFA was undertaken to support the dimensional structure identified by AISP. EFA was conducted using the principal-factor method and oblique (oblimin) rotation. We also examined the standardized residuals of item-pair correlations.

### Known group analysis

Scales that demonstrated sufficient scaling properties were examined across subgroups hypothesized to differ in their level of health and well-being: being a carer and having a long-term illness. The LSS was calculated by assigning a numerical value to each response level (1 for least severe to 5 for the most severe response), and these are summed across the items of the scales. The scales were transformed into a 0 to100 score to allow for comparability across scales as well as with the EQ VAS. The LSS were compared across these subgroups using non-parametric rank-sum tests. Multivariate linear regression was used to examine these variables controlling for age, gender and race.

### Robustness of results

All scaling analyses were stratified by country, carer status, having a long-term condition, gender, and age groups. The sample size of all subgroups exceeded *n* = 500, therefore the standard rules for determining rest-score groups were consistent.

## Results

In total, 3340 respondents were included in the psychometric study from the US (*n* = 903), AUS (*n* = 514) and the UK (*n* = 1923). Just over half were women and the average age was 50 years (Table 1). The majority (73.5%) self-reported having one or more long-term condition(s). The average EQ VAS (66.55) was low as compared to general population norms [34–36]; nearly 30% self-identified as a carer.

**Table 1:** Characteristics of the study sample

	US		AUS		UK		Total	
	*N*, mean	(%, SD)	*N*, mean	(%, SD)	*N*, mean	(%, SD)	*N*, mean	(%, SD)
	903	(27.04%)	514	(15.39%)	1923	(57.57%)	3340	
Women	436	(48.28%)	308	(59.92%)	1097	(57.05%)	1841	(55.12%)
Have a long-term condition	623	(68.99%)	374	(72.76%)	1460	(75.92%)	2457	(73.56%)
Non-White	133	(14.73%)	90	(17.51%)	169	(8.79%)	392	(11.74%)
Age	53.81	(17.46)	49.88	(16.95)	48.58	(18.80)	50.19	(18.30)
EQ VAS	72.25	(19.36)	64.98	(22.92)	64.43	(24.30)	66.55	(23.14)
EQ-5D-5L Level Sum Score	82.90	(17.03)	77.13	(18.75)	77.78	(20.85)	79.05	(19.71)
EQ-5D-5L US Value Set	0.762	(0.251)	0.672	(0.296)	0.682	(0.327)	0.702	(0.308)

### AISP and monotonicity

Table 2 shows the AISP results with the lower bound set from 0.1 to 0.6. When the lower bound was set at 0.448, the physical items (*n* = 3) and psychosocial items (*n* = 6) were placed on different scales (Table 2). We tested AISP using the items get around inside, get around outside, concentrating and thinking clearly as individual (non-composite) items, and results did not substantively differ from AISP that used the composite items. Therefore, we used the composite items in further analyses.

**Table 2:** Mokken Scaling Automatic Item Selection Process Results

	Lower bound H_S_: HWB-S					
	0.1	0.2	0.3	0.4	0.5	0.6
Anxiety	1	1	1	1	2	2
Sad	1	1	1	1	2	2
Fatigue	1	1	1	1	2	2
Loneliness	1	1	1	1	2	2
Concentrating/thinking	1	1	1	1	2	2
No control (no def)	1	1	1	1	2	2
Pain (severity)	1	1	1	1	1	0
Daily activities	1	1	1	1	1	1
Mobility (inside/outside)	1	1	1	1	1	1

0 Unscalable, 1 Belonging to the first scale, 2 Belonging to a second scale

When the EQ-HWB-S was modeled as a single scale, its H_S_ indicated a strong scale at 0.561. The items “pain severity”, “daily activities” and “mobility” were moderately scalable (H_i_ 0.396–0.496) while the rest of the items were strongly scalable (H_i_ 0.590–0.626). When modeled as two subscales, the physical (H_S_ = 0.683) and psychosocial (H_S_ = 0.713) components had stronger scalability and strong H_i_ for all items (Table 3).

**Table 3:** Scalability, monotonicity and MIIO results for the EQ-HWB-S, single scale and two subscales

	Mean	Scalability		Monotonicity			MIIO		
		H_S_	(SE)	AC	VI	Crit	AC	VI	Crit
Anxiety	2.450	0.610	(0.008)	105	0	0	55	4	158
Sad	2.523	0.618	(0.008)	93	0	0	55	4	125
Fatigue	2.924	0.590	(0.009)	112	0	0	51	1	42
Loneliness	2.260	0.591	(0.009)	93	0	0	55	4	192
Concentrating/thinking	2.674	0.608	(0.009)	112	0	0	55	2	77
No control (no def)	2.146	0.626	(0.008)	128	0	0	55	7	245*
Pain severity	2.212	0.396	(0.013)	112	0	0	56	16	385*
Daily activities	1.931	0.496	(0.012)	98	0	0	56	3	75
Mobility	1.821	0.446	(0.012)	120	0	0	56	1	45
Scale H		0.561	(0.008)						
Anxiety	2.450	0.737	(0.007)	74	0	0	30	0	0
Sad	2.523	0.742	(0.007)	92	0	0	31	0	0
Fatigue	2.924	0.655	(0.010)	84	0	0	31	0	0
Loneliness	2.260	0.714	(0.008)	62	0	0	31	0	0
Concentrating/thinking	2.674	0.713	(0.008)	84	0	0	30	0	0
No control (no def)	2.146	0.716	(0.009)	112	0	0	31	0	0
Scale H		0.713	(0.007)						
Pain severity	2.212	0.590	(0.016)	40	0	0	6	2	322*
Daily activities	1.931	0.724	(0.010)	40	0	0	6	1	215
Mobility	1.821	0.722	(0.010)	50	0	0	6	1	148
Scale H		0.680	(0.011)						

*MIIO* manifest item invariant odering, *H*_*S*_ Loevinger’s scaling coefficients H for scale, *SE* standard error, *AC* active pairs, *VI* violations, *Crit* crit coefficient

*Items which were suggested for removal during backward item selection

**Fig. 2:**
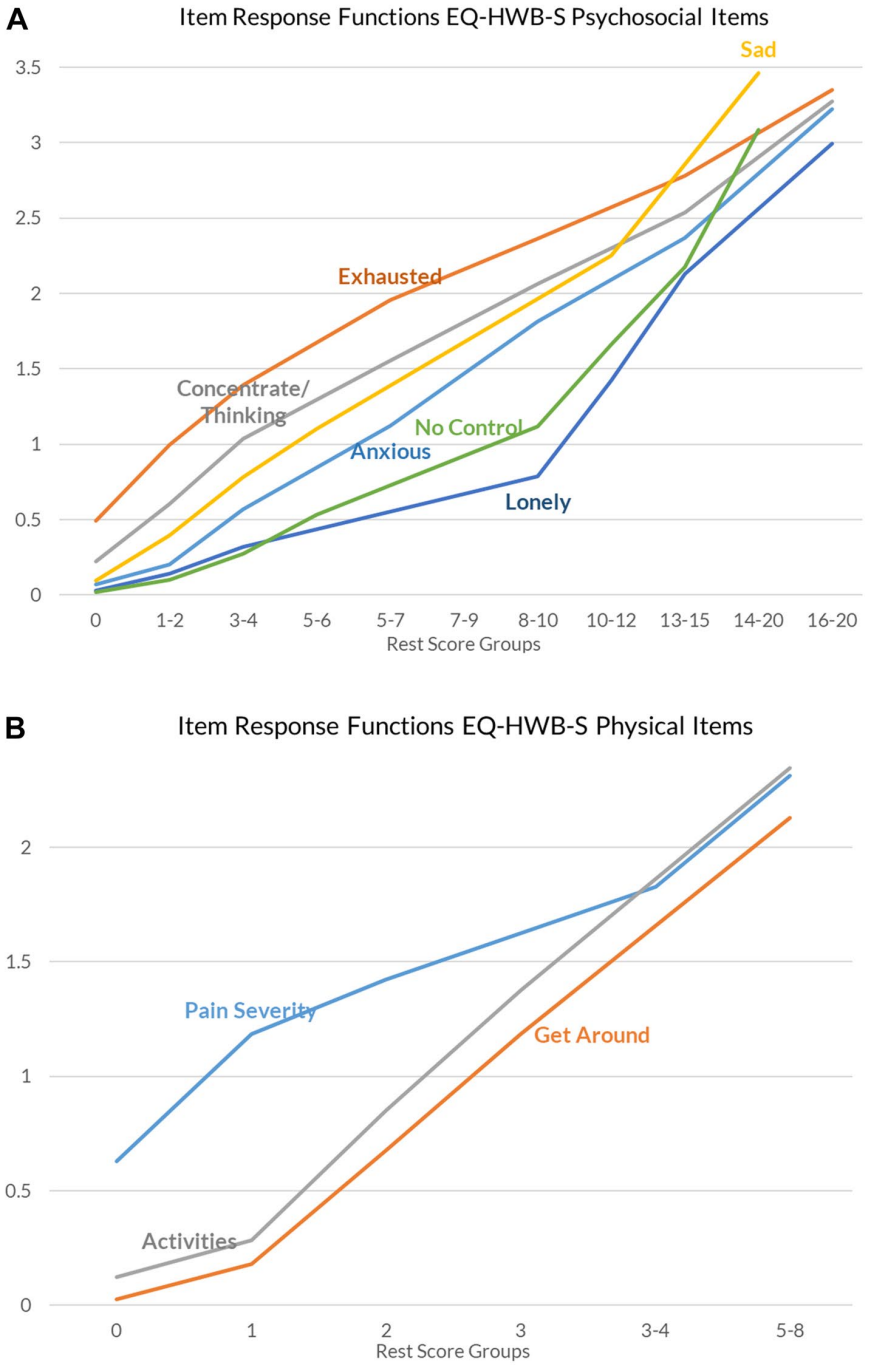
Item response functions for the EQ-HWB-S subscales

No violations of monotonicity were identified: Crit values of all items were zero, showing no misfit of the MHM. We used selected item-pair results from the ‘check.restscore’ function to visualize the IRF of multiple items in one figure (Fig. 2). The IRF figures for the EQ-HWB-S subscales visually illustrate that as the rest score increased, the sum of items’ ISRFs did not decrease. The full set of ISRF and IRF figures available in Appendix D.

### Fit of MIIO

As a single scale, violations of MIIO were observed for every item, with backward item selection removing the No Control (7 violations, Crit 245) and Pain (16 violations, Crit 385) items (Table 3). No violations were observed for the psychosocial subscale but violations were found for all items of the physical subscale, seemingly due to the Pain item (2 violations, Crit 322). H^T^ indicated that items could not be ordered or the order to have low accuracy for all scales and subgroups (Table 4).

**Table 4:** EQ-HWB-S Scale H coefficients stratified across subgroups

	*n*	Single scale			Psychosocial subscale			Physical subscale		
		H_S_	(SE)	H^T^	H_S_	(SE)	H^T^	H_S_	(SE)	H^T^
Complete data	3268	0.561	(0.008)	0.219	0.713	(0.007)	0.209	0.683	(0.011)	0.183
US	872	0.575	(0.016)	0.202	0.723	(0.014)	0.232	0.665	(0.024)	0.317
Australia	514	0.553	(0.020)	0.232	0.689	(0.019)	0.197	0.727	(0.023)	0.393
UK	1882	0.546	(0.010)	0.229	0.703	(0.009)	0.203	0.678	(0.014)	0.099
Women	1805	0.554	(0.010)	0.279	0.716	(0.009)	0.265	0.684	(0.015)	0.198
Men	1460	0.567	(0.012)	0.147	0.702	(0.011)	0.147	0.681	(0.016)	0.169
Long-term condition	2392	0.542	(0.009)	0.187	0.705	(0.008)	0.219	0.654	(0.013)	0.160
No long-term condition	870	0.543	(0.017)	0.377	0.694	(0.016)	0.182	0.561	(0.033)	0.310
Carer	893	0.553	(0.015)	0.213	0.682	(0.014)	0.171	0.691	(0.020)	0.150
Not a carer	2366	0.559	(0.009)	0.221	0.723	(0.008)	0.228	0.675	(0.013)	0.195
Age ≤ 35	885	0.561	(0.015)	0.484	0.683	(0.014)	0.194	0.596	(0.024)	0.103
Age 36 to 50	759	0.671	(0.016)	0.282	0.694	(0.022)	0.185	0.559	(0.015)	0.175
Age 51 to 65	802	0.587	(0.016)	0.197	0.696	(0.015)	0.243	0.756	(0.018)	0.354
Age > 65	814	0.528	(0.018)	0.162	0.620	(0.018)	0.255	0.703	(0.021)	0.185

*H*_*S*_ Loevinger’s scaling coefficients H for scale, *SE* standard error, *H*^*T*^ coefficient calculated without exclusion due to backward item selection

### Stratified analysis across subgroups

Scaling coefficients were strong to very strong and did not differ substantively across most of the sub-populations (country, gender, age categories, having a self-reported long-term health condition, being a carer, Table 4).

As a single scale, statistically significant violations of monotonicity were found for those with long-term conditions (mobility), and the UK population (mobility and daily activities), indicating that those items did not discriminate well for those sub-populations. No violations of monotonicity were found for the subscales across all subgroups. See Appendices E1 and E2 for detailed monotonicity results.

In terms of MIIO, the items of the single scale for those without a long-term condition and the physical subscale for subgroups US, AUS, those without a long-term condition and aged 51 to 65 could be ordered but with low accuracy. Only the single scale for the subgroup aged ≤ 35 had an H^T^ larger than 0.4, indicating moderate accuracy of item order.

Lastly, we used the Australian data to examine the items “feel sad” and “feel depressed”: the items were combined in the same way as concentrating/thinking and mobility inside/outside. Models using the composite sad/depressed item were similar to models using the sad item, with slightly better scaling properties for the combined item.

### EFA results

Eigenvalues were larger than 1 for two factors. With an Eigenvalue value of 4.80, a dominant first factor was extracted while the Eigenvalue for factor 2 was just over the threshold of 1.0 (Appendix F). Standardized residual correlations tended to be large for item pairs that include the physical items for the 1-factor solution, while all but one (item pair “pain” and “exhausted”) standardized residuals were adequately small for the 2-factor solution.

### Descriptive analysis of the EQ-HWB-S LSS

**Fig. 3:**
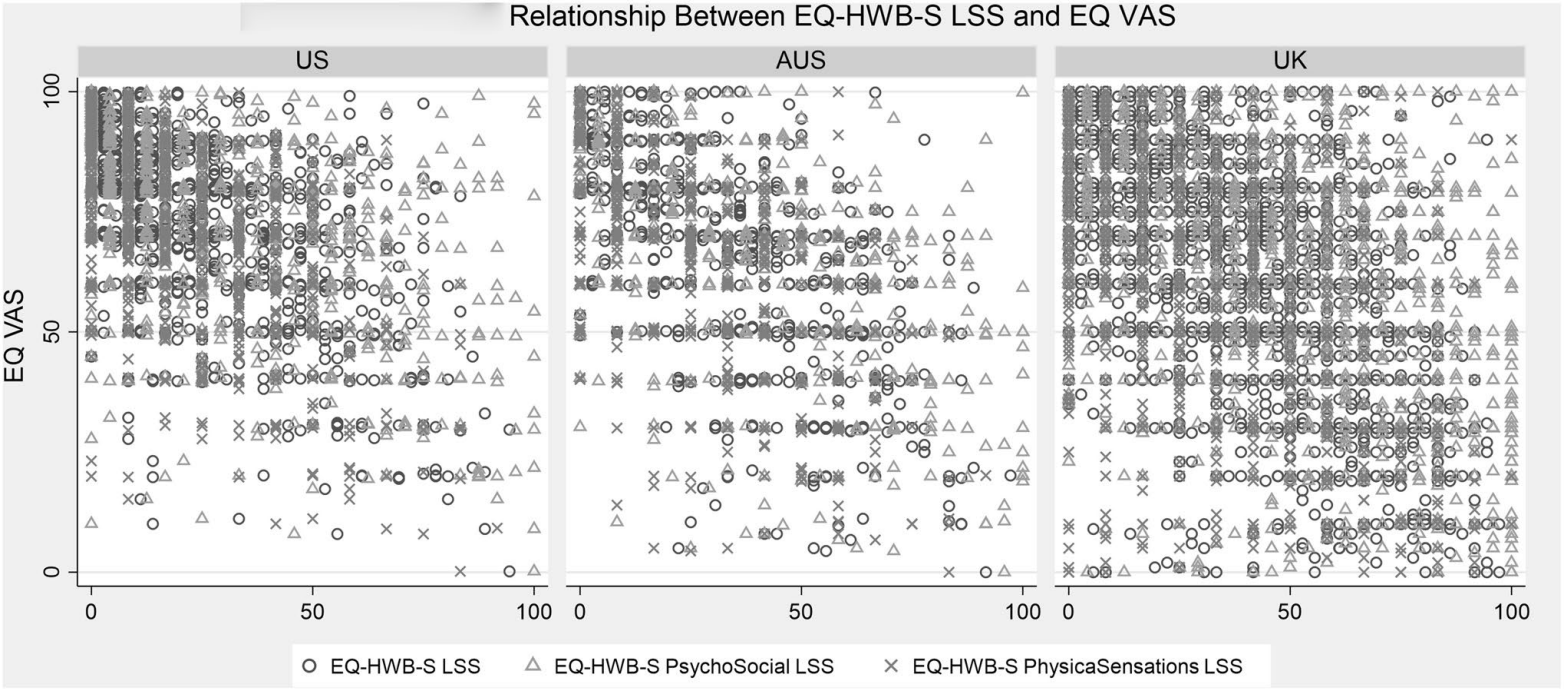
Relationship between EQ-HWB-S level summary scores and EQ VAS

The reliability was excellent for the single scale and psychosocial subscale (alpha and lambda > 0.9) and good for the physical subscale (alpha and lambda > 0.8). The LSS was calculated for all 9 items of the EQ-HWB-S, the 6 psychosocial items and the 3 physical sensation items: all were moderately correlated with EQ VAS with rho of − 0.55 to − 0.62 (Fig. 3). Distribution of items responses and scale/subscale scores did not reveal problematic skews or irregular response patterns.

Only 176 (5.41%) respondents reported the lowest score (or no problems on all items), at the scale level, while 346 (10.53%) reported the lowest score for the psychosocial subscale and 676 (20.54%) for the physical sensations subscale. No respondents reported the highest possible score on the full EQ-HWB-S LSS while 79 (2.40%) reported the highest score on the psychosocial subscale and 3 (0.09%) on the physical sensations subscale. Of those reporting the top score (11111) on the EQ-5D-5L (589, 17.85%), 159 (27.27%) also reported the lowest EQ-HWB-S, 188 (32.14%) psychosocial, and 415 (71.06%) physical sensations LSS, showing a similarity between the physical subscale and the EQ-5D-5L. Of those without a long-term condition, 110 (12.64%) reported the lowest score for the overall LSS and 156 (17.91%) on the psychosocial subscale. However, 383 (43.87%) reported the lowest score on the physical sensations subscale.

**Fig. 4:**
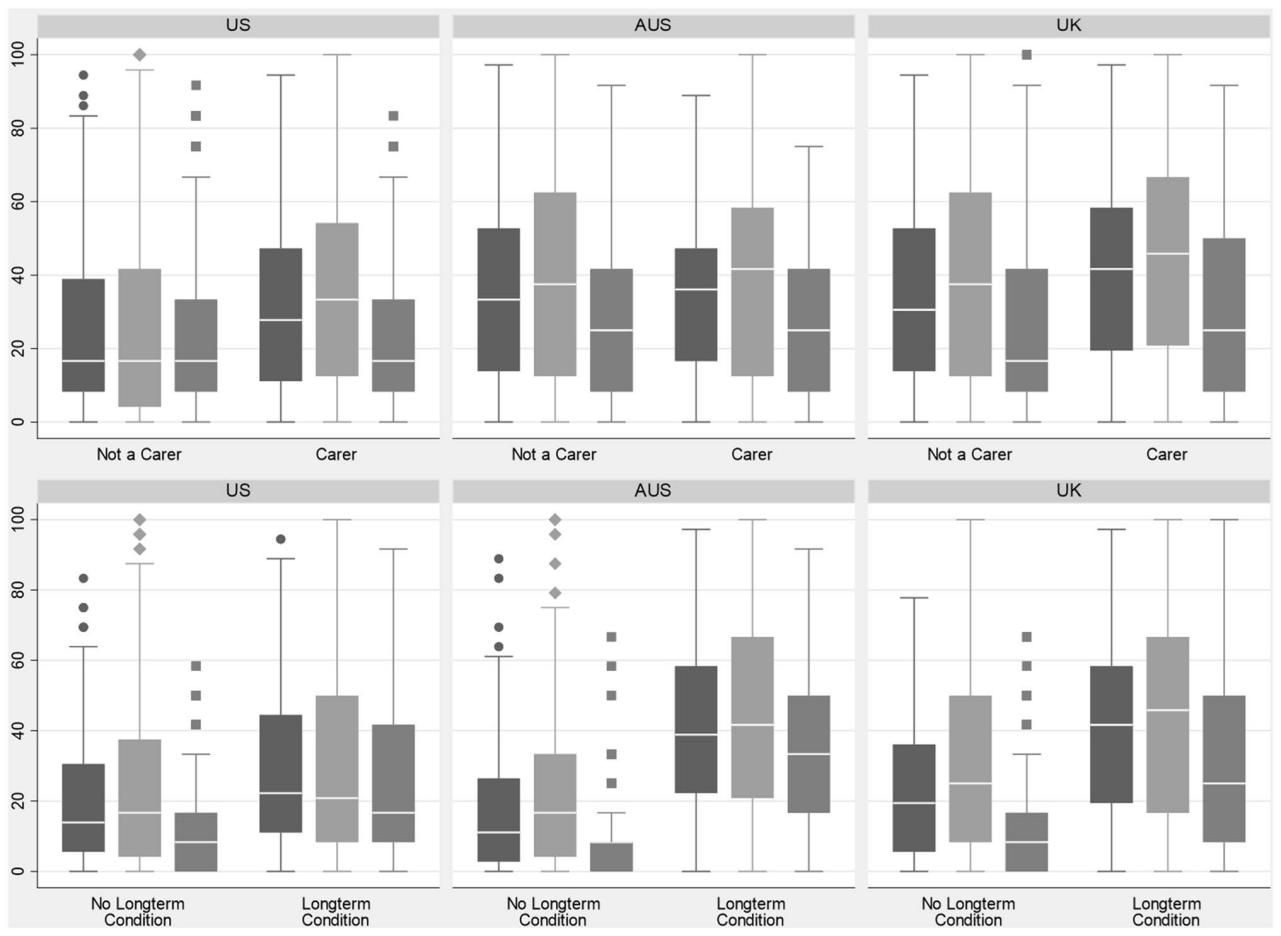
EQ-HWB-S level summary scores across known groups

The differences in LSS across carers/non-carers, those with and those without long-term conditions were statistically significant (Table 5) with higher full scale and subscales scores (reporting more symptoms and problems) for those who are carers and those with long-term condition(s). These results did not differ substantively across country subgroups (Fig. 4) nor after adjusting for age, gender and race (results not shown).

**Table 5:** EQ-HWB-S Level Summary Scores Across Known Groups

	*n*	EQ-HWB-S LSS		EQ-HWB-S psychosocial LSS		EQ-HWB-S physical sensations l LSS	
		Median	(IQR)	Median	(IQR)	Median	(IQR)
Total	3255	30.56	(11.11–50.00)	33.33	(12.50–58.33)	16.67	(8.33–41.67)
Not carer	2382	27.78	(11.11–50.00)	29.17	(8.33–58.33)	16.67	(8.33–41.67)
Carer	900	36.11	(16.67–55.56)	41.67	(16.67–62.50)	25.00	(8.33–41.67)
*P*-value (Wilcoxon)		< 0.0001		< 0.0001		< 0.0001	
No longterm condition	873	16.67	(5.56–33.33)	20.83	(4.17–45.83)	8.33	(0.00–16.67)
Longterm condition	2412	36.11	(16.67–55.56)	37.50	(12.50–62.50)	25.00	(8.33–41.67)
*P*-value (Wilcoxon)		< 0.0001		< 0.0001		< 0.0001

## Discussion

Items of the EQ-HWB-S formed a strong Mokken scale, giving support for use of LSS either at the overall scale or subscale levels. Scalability was strong both for the single scale and the two subscales (6 psychosocial and 3 physical items). No violations of manifest monotonicity were found for the combined datasets, suggesting that the LSS ordered respondents along the latent trait. Therefore, the LSS of the EQ-HWB-S, scale or subscales, can generally order respondents along the latent trait: as *θ* increases, respondents are more likely to choose increasingly more severe response levels. These results empirically demonstrate that the summary score ordered respondents by their levels of health and well-being. Scaling results were robust across sub-populations (not weaker for healthier subgroups as previously found for the EQ-5D-5L [8]). Overall, scalability results are comparable with previous Mokken investigations of the EQ-5D-5L and the SF-36 [8, 37].

Although no violations of monotonicity were found for subscales across all investigated subgroups, violations were identified for the single scale for sub-populations, showing some items (mobility, daily activities) to not discriminate well for respondents from the UK and those with a long-term health condition. Items could not be ordered along the LSS of these scales.

The early development phases of the EQ-HWB included qualitative research and stakeholder input [2, 5] which largely informed the first psychometric investigations and theoretical dimensional structure of candidate items (see Peasgood et al. for a full exploration of that dimensional structure [1, 3]). While the theory driven model informs the conceptual model for the EQ-HWB, our goal was to provide support for a pragmatic method of summarizing the short-form using a data-driven approach. When the theoretical dimensional structure was not imposed on the data, EFA of the full set of 60 + EQ-HWB candidate items identified only three factors [3], which is similar to our finding that the 9-item instrument can be described using two subscales. This, along with the sufficiently large sample size and robustness of results across subgroups of our study lends confidence that the strong scalability was not a spurious finding. However, AISP may yield a dimensionality structure differing from more conventional techniques, such as EFA: a simulation study found that AISP may not adequately identify appropriate number of dimensions when the factors are strongly correlated [38].

Adding EFA in this investigation provides additional support for the dimensionality as EFA and AISP revealed similar structures in the data: a two-factor solution with a physical and a psychosocial domain had the strongest scalability and most reasonable residual correlation results, and therefore would best describe the structure of the 9 items. Yet, given the dominant first factor (based on eigenvalue, factor loadings and scalability results), adopting a simpler solution for scoring the 9 items may be acceptable. These results may indicate a second-order structure, with a physical and a psychosocial domain both belonging to an overall “well-being” scale. New methods were recently developed to test multidimensionality and higher-order monotone factor models [39]. However, this new methodology has only been developed for binary data, needs further refinement and testing, and is not yet available in software. Clarifying this higher-order structure should be conducted in future studies.

We interpret the results of the Mokken analysis and supporting EFA that using the physical and psychosocial subscales would best reflect the structure underlying the EQ-HWB-S, while using a single score would also be acceptable. Using subscales may be more sensitive to specific patient populations and interventions. Practically, a single score is a powerful generic measure which allows for comparison across patient groups, but loses dimension specificity and interpreting at the subscale level. The physical subscale has slightly poorer scalability and MIIO results than the psychosocial subscale.

Although we were limited in known group comparisons as the dataset was not originally collected for such analyses, we were able to show the EQ-HWB-S scale and subscales differentiated across important subgroups such as those with and without long-term illness. Although scores were statistically different across age groups, older age groups tended to have better EQ-HWB-S scores than younger age groups, as was the case for the EQ-5D-5L health profile and EQ VAS. The counterintuitive results found for age reflect some other recent findings in population level surveys [40, 41].

While we found preliminary support for an LSS approach based on empirical analysis of EQ-HWB-S data, the original scale is conceptually multidimensional and an analysis including additional items (e.g. from other measures of well-being) would provide greater clarity on scalability. Further research is needed as more data of the EQ-HWB become available. Another limitation of this study is that the datasets did not contain the similar wordings to the current experimental EQ-HWB-S instrument for three items: the analysis must be repeated using the most recent version of the EQ-HWB to obtain a more accurate assessment of scaling properties of the instrument. This is especially true for the “sad” item as Australia was the only sample which had sufficient data to examine the combined sad/depressed item. A third limitation is that only English versions of the EQ-HWB-S were analyzed: non-English EQ-HWB versions were included as a part of the psychometric study and extending these analyses to other languages and countries would be necessary. Lastly, given the strong scalability findings for the EQ-HWB (with items conceptually measuring different concepts), it is possible that similar groupings of health and well-being domains were observed as the survey sampled particular condition and population groups. This pattern (and the appropriateness of using the LSS) may differ for other patient populations. These results should be confirmed in a broader set of patient populations to examine whether two subscales for health and well-being can be justified more broadly.

## Conclusion

LSS is a promising approach to scoring the psychosocial and physical subscales of the EQ-HWB-S. However, a study including a more representative sample and using the most up-to-date version of the EQ-HWB-S items is needed to further support LSS for the EQ-HWB-S.

### Supplementary Information

Below is the link to the electronic supplementary material.

Supplementary file1 (R 20 kb)

Supplementary file2 (DOCX 12 kb)

Supplementary file3 (DOCX 13 kb)

Supplementary file4 (JPG 927 kb)

Supplementary file5 (DOCX 20 kb)

Supplementary file6 (DOCX 15 kb)

## Data Availability

Contact the primary author regarding data use/data sharing.
