# Supplementary material for: Scoring the EQ-HWB-S: can we do it without value sets? A non-parametric item response theory analysis
Source: Qual Life Res. 2024 Feb 21;33(5):1211–22. doi: 10.1007/s11136-024-03601-7 (PMC11045574; doi:10.1007/s11136-024-03601-7)
Supplement: Supplementary file 2 — Supplementary file2 (DOCX 12 kb) [file 11136_2024_3601_MOESM2_ESM.docx]

| **Appendix B: EQ-HWB Item Description and Abbreviation** | |
| --- | --- |
| **EQ-HWB Item Short Description** | **EQ-HWB Item Reference in Text** |
| Felt anxious | Anxiety |
| Felt sad | Sad |
| Felt exhausted | Fatigue |
| Felt lonely | Loneliness |
| Hard to concentrate | Concentrating |
| Trouble thinking clearly | Thinking |
| Felt in control of day-to-day life (with a definition) | Control (def) |
| Felt no control over day-to-day life (no definition) | No Control (no def) |
| Physical pain (severity response choices) | Pain (severity) |
| able to do day-to-day activities | Daily Activities |
| Get around inside your home | Mobility (inside) |
| Get around outside | Mobility (outside) |
