# Supplementary material for: Scoring the EQ-HWB-S: can we do it without value sets? A non-parametric item response theory analysis
Source: Qual Life Res. 2024 Feb 21;33(5):1211–22. doi: 10.1007/s11136-024-03601-7 (PMC11045574; doi:10.1007/s11136-024-03601-7)
Supplement: Supplementary file 3 — Supplementary file3 (DOCX 13 kb) [file 11136_2024_3601_MOESM3_ESM.docx]

| **Appendix C: Major Changes in Post-Dataset Collection Modifications to Wording of EQ-HWB Items** | | |
| --- | --- | --- |
|  | **Wording in the experimental EQ-HWB-S** | **Wording in the psychometric survey** |
| **Major Changes** | How difficult was it for you to get around inside and outside *(using, for example, walking stick, frame or wheelchair if you usually use them)*? [No difficulty… unable] | 2 items: ⮞How well were you able to get around outside? ⮞How well were you able to get around inside your home? [No difficulty… unable] |
|  | I had trouble concentrating or thinking clearly | 2 items: ⮞I had trouble thinking clearly  ⮞I found it hard to concentrate |
|  | I felt sad or depressed | I felt sad |
| **Minor to No changes** | I felt I had no control over my day-to-day life *(had the choice to do things or have things done for you as you like and when you wanted)* | 3 items: ⮞I felt I had no control over my day to day life ⮞I felt in control of my day to day life ⮞I felt in control of my day to day life By ‘control over day to day life’ we mean having the choice to do things or have things done for you as you like and when you want. |
|  | How difficult was it for you to do day-to-day activities *(for example, working, shopping, housework)*? [No difficulty… unable] | How well were you able to do your day to day activities (e.g. working, shopping, travelling)? [No difficulty… unable] |
|  | I felt anxious | Identical |
|  | I felt exhausted | Identical |
|  | I felt lonely | Identical |
|  | Please tick one box to describe your experience in the last 7 days: I had no physical pain… I had very severe physical pain | Identical |
