# Supplementary figures and images for: Scoring the EQ-HWB-S: can we do it without value sets? A non-parametric item response theory analysis
Source: Qual Life Res. 2024 Feb 21;33(5):1211–22. doi: 10.1007/s11136-024-03601-7 (PMC11045574; doi:10.1007/s11136-024-03601-7)

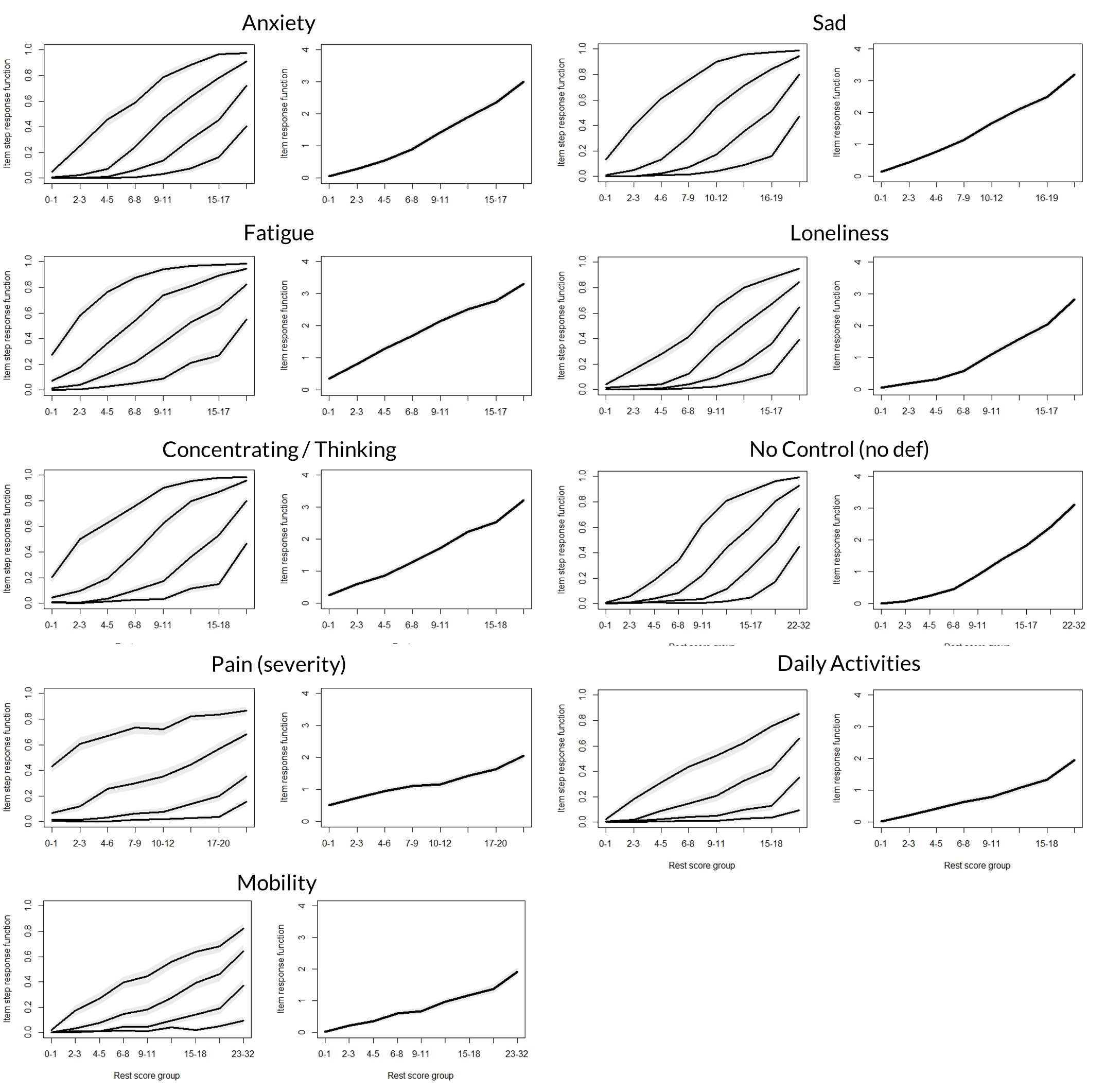

Supplement: Supplementary file 4 — Supplementary file4 (JPG 927 kb) [file 11136_2024_3601_MOESM4_ESM.jpg]
