# Supplementary material for: Scoring the EQ-HWB-S: can we do it without value sets? A non-parametric item response theory analysis
Source: Qual Life Res. 2024 Feb 21;33(5):1211–22. doi: 10.1007/s11136-024-03601-7 (PMC11045574; doi:10.1007/s11136-024-03601-7)
Supplement: Supplementary file 5 — Supplementary file5 (DOCX 20 kb) [file 11136_2024_3601_MOESM5_ESM.docx]

| **Appendix E1: EQ-HWB-S Scale Monotonicity Results Across Subgroups** | | | | | | | | | | | | | | | | |
| --- | --- | --- | --- | --- | --- | --- | --- | --- | --- | --- | --- | --- | --- | --- | --- | --- |
|  | **US** | | | | **Australia** | | | | **UK** | | | | **All Data** | | | |
|  | #VI | #VI/#AC | ZSig | Crit | #VI | #VI/#AC | ZSig | Crit | #VI | #VI/#AC | ZSig | Crit | #VI | #VI/#AC | ZSig | Crit |
| Anxious | 0 | 0 | 0 | 0 | 0 | 0 | 0 | 0 | 0 | 0 | 0 | 0 | 0 | 0 | 0 | 0 |
| Sad | 0 | 0 | 0 | 0 | 0 | 0 | 0 | 0 | 0 | 0 | 0 | 0 | 0 | 0 | 0 | 0 |
| Exhausted | 0 | 0 | 0 | 0 | 2 | 0.02 | 0 | 3 | 0 | 0 | 0 | 0 | 0 | 0 | 0 | 0 |
| Lonely | 0 | 0 | 0 | 0 | 0 | 0 | 0 | 0 | 0 | 0 | 0 | 0 | 0 | 0 | 0 | 0 |
| Concentrate/Thinking | 0 | 0 | 0 | 0 | 0 | 0 | 0 | 0 | 0 | 0 | 0 | 0 | 0 | 0 | 0 | 0 |
| No Control (no def) | 0 | 0 | 0 | 0 | 0 | 0 | 0 | 0 | 0 | 0 | 0 | 0 | 0 | 0 | 0 | 0 |
| Pain Severity | 0 | 0 | 0 | 0 | 0 | 0 | 0 | 0 | 1 | 0.01 | 0 | 12 | 0 | 0 | 0 | 0 |
| Activities | 2 | 0.03 | 0 | 11 | 3 | 0.04 | 0 | 14 | **1** | **0.01** | **1** | **23** | 0 | 0 | 0 | 0 |
| Get Around | 0 | 0 | 0 | 0 | 2 | 0.04 | 0 | 13 | **1** | **0.01** | **1** | **25** | 0 | 0 | 0 | 0 |
|  | **Long-term Condition** | | | | **No Long-term Condition** | | | | **Carer** | | | | **Not a Carer** | | | |
| Anxious | 0 | 0 | 0 | 0 | 0 | 0 | 0 | 0 | 0 | 0 | 0 | 0 | 0 | 0 | 0 | 0 |
| Sad | 0 | 0 | 0 | 0 | 0 | 0 | 0 | 0 | 1 | 0.01 | 0 | -5 | 0 | 0 | 0 | 0 |
| Exhausted | 0 | 0 | 0 | 0 | 0 | 0 | 0 | 0 | 0 | 0 | 0 | 0 | 0 | 0 | 0 | 0 |
| Lonely | 0 | 0 | 0 | 0 | 0 | 0 | 0 | 0 | 0 | 0 | 0 | 0 | 0 | 0 | 0 | 0 |
| Concentrate/Thinking | 0 | 0 | 0 | 0 | 0 | 0 | 0 | 0 | 1 | 0.01 | 0 | 5 | 0 | 0 | 0 | 0 |
| No Control (no def) | 0 | 0 | 0 | 0 | 0 | 0 | 0 | 0 | 0 | 0 | 0 | 0 | 0 | 0 | 0 | 0 |
| Pain Severity | 0 | 0 | 0 | 0 | 2 | 0.03 | 0 | 30 | 4 | 0.04 | 0 | 17 | 2 | 0.02 | 0 | 10 |
| Activities | 0 | 0 | 0 | 0 | 1 | 0.02 | 0 | 11 | 0 | 0 | 0 | 0 | 0 | 0 | 0 | 0 |
| Get Around | **1** | **0.01** | **1** | **23** | 1 | 0.02 | 0 | 9 | 0 | 0 | 0 | 0 | 0 | 0 | 0 | 0 |
|  | **Age ≤ 35** | | | | **Age 36 to 50** | | | | **Age 51 to 65** | | | | **Age > 65** | | | |
| Anxious | 0 | 0 | 0 | 0 | 1 | 0.01 | 0 | -6 | 0 | 0 | 0 | 0 | 0 | 0 | 0 | 0 |
| Sad | 0 | 0 | 0 | 0 | 0 | 0 | 0 | 0 | 0 | 0 | 0 | 0 | 0 | 0 | 0 | 0 |
| Exhausted | 0 | 0 | 0 | 0 | 0 | 0 | 0 | 0 | 0 | 0 | 0 | 0 | 0 | 0 | 0 | 0 |
| Lonely | 0 | 0 | 0 | 0 | 0 | 0 | 0 | 0 | 0 | 0 | 0 | 0 | 0 | 0 | 0 | 0 |
| Concentrate/Thinking | 0 | 0 | 0 | 0 | 0 | 0 | 0 | 0 | 0 | 0 | 0 | 0 | 0 | 0 | 0 | 0 |
| No Control (no def) | 1 | 0.01 | 0 | 2 | 0 | 0 | 0 | 0 | 0 | 0 | 0 | 0 | 0 | 0 | 0 | 0 |
| Pain Severity | 0 | 0 | 0 | 0 | 1 | 0.01 | 0 | 8 | 0 | 0 | 0 | 0 | 1 | 0.01 | 0 | 13 |
| Activities | 0 | 0 | 0 | 0 | 1 | 0.01 | 0 | 3 | 0 | 0 | 0 | 0 | 0 | 0 | 0 | 0 |
| Get Around | 1 | 0.01 | 0 | 4 | 0 | 0 | 0 | 0 | 0 | 0 | 0 | 0 | 0 | 0 | 0 | 0 |
| **Bold**: Statistically significant violations | | | | | | | | | | | | | | | | |
| #VI: number of violations found | | | | | | | | | | | | | | | | |
| #VI/#AC: average number of violations for all active pairs of items compared | | | | | | | | | | | | | | | | |
| ZSig: Number of statistically significant violations | | | | | | | | | | | | | | | | |
| Crit: Critical Value for the Violations | | | | | | | | | | | | | | | | |

| **Appendix E2: EQ-HWB-S Subscale Monotonicity Results Across Subgroups** | | | | | | | | | | | | | | | | |
| --- | --- | --- | --- | --- | --- | --- | --- | --- | --- | --- | --- | --- | --- | --- | --- | --- |
|  | **US** | | | | **Australia** | | | | **UK** | | | | **All Data** | | | |
| **Psychosocial Subscale** | #VI | #VI/#AC | ZSig | Crit | #VI | #VI/#AC | ZSig | Crit | #VI | #VI/#AC | ZSig | Crit | #VI | #VI/#AC | ZSig | Crit |
| Anxious | 0 | 0 | 0 | 0 | 0 | 0 | 0 | 0 | 0 | 0 | 0 | 0 | 0 | 0 | 0 | 0 |
| Sad | 0 | 0 | 0 | 0 | 0 | 0 | 0 | 0 | 0 | 0 | 0 | 0 | 0 | 0 | 0 | 0 |
| Exhausted | 0 | 0 | 0 | 0 | 1 | 0.01 | 0 | 0 | 0 | 0 | 0 | 0 | 0 | 0 | 0 | 0 |
| Lonely | 0 | 0 | 0 | 0 | 0 | 0 | 0 | 0 | 0 | 0 | 0 | 0 | 0 | 0 | 0 | 0 |
| Concentrate/Thinking | 0 | 0 | 0 | 0 | 0 | 0 | 0 | 0 | 0 | 0 | 0 | 0 | 0 | 0 | 0 | 0 |
| No Control (no def) | 0 | 0 | 0 | 0 | 1 | 0.01 | 0 | -10 | 0 | 0 | 0 | 0 | 0 | 0 | 0 | 0 |
| **Physical Subscale** |  |  |  |  |  |  |  |  |  |  |  |  |  |  |  |  |
| Pain Severity | 0 | 0 | 0 | 0 | 0 | 0 | 0 | 0 | 0 | 0 | 0 | 0 | 0 | 0 | 0 | 0 |
| Activities | 0 | 0 | 0 | 0 | 0 | 0 | 0 | 0 | 0 | 0 | 0 | 0 | 0 | 0 | 0 | 0 |
| Get Around | 0 | 0 | 0 | 0 | 0 | 0 | 0 | 0 | 0 | 0 | 0 | 0 | 0 | 0 | 0 | 0 |
|  | **Long-term Condition** | | | | **No Long-term Condition** | | | | **Carer** | | | | **Not a Carer** | | | |
| **Psychosocial Subscale** |  |  |  |  |  |  |  |  |  |  |  |  |  |  |  |  |
| Anxious | 0 | 0 | 0 | 0 | 0 | 0 | 0 | 0 | 0 | 0 | 0 | 0 | 0 | 0 | 0 | 0 |
| Sad | 0 | 0 | 0 | 0 | 0 | 0 | 0 | 0 | 0 | 0 | 0 | 0 | 0 | 0 | 0 | 0 |
| Exhausted | 0 | 0 | 0 | 0 | 0 | 0 | 0 | 0 | 0 | 0 | 0 | 0 | 0 | 0 | 0 | 0 |
| Lonely | 0 | 0 | 0 | 0 | 0 | 0 | 0 | 0 | 0 | 0 | 0 | 0 | 0 | 0 | 0 | 0 |
| Concentrate/Thinking | 0 | 0 | 0 | 0 | 0 | 0 | 0 | 0 | 0 | 0 | 0 | 0 | 0 | 0 | 0 | 0 |
| No Control (no def) | 0 | 0 | 0 | 0 | 0 | 0 | 0 | 0 | 0 | 0 | 0 | 0 | 0 | 0 | 0 | 0 |
| **Physical Subscale** |  |  |  |  |  |  |  |  |  |  |  |  |  |  |  |  |
| Pain Severity | 0 | 0 | 0 | 0 | 0 | 0 | 0 | 0 | 0 | 0 | 0 | 0 | 0 | 0 | 0 | 0 |
| Activities | 0 | 0 | 0 | 0 | 0 | 0 | 0 | 0 | 0 | 0 | 0 | 0 | 0 | 0 | 0 | 0 |
| Get Around | 0 | 0 | 0 | 0 | 0 | 0 | 0 | 0 | 0 | 0 | 0 | 0 | 0 | 0 | 0 | 0 |
|  | **Age ≤ 35** | | | | **Age 36 to 50** | | | | **Age 51 to 65** | | | | **Age > 65** | | | |
| **Psychosocial Subscale** |  |  |  |  |  |  |  |  |  |  |  |  |  |  |  |  |
| Anxious | 0 | 0 | 0 | 0 | 0 | 0 | 0 | 0 | 0 | 0 | 0 | 0 | 0 | 0 | 0 | 0 |
| Sad | 1 | 0.01 | 0 | -7 | 0 | 0 | 0 | 0 | 0 | 0 | 0 | 0 | 0 | 0 | 0 | 0 |
| Exhausted | 0 | 0 | 0 | 0 | 0 | 0 | 0 | 0 | 0 | 0 | 0 | 0 | 0 | 0 | 0 | 0 |
| Lonely | 0 | 0 | 0 | 0 | 0 | 0 | 0 | 0 | 0 | 0 | 0 | 0 | 0 | 0 | 0 | 0 |
| Concentrate/Thinking | 0 | 0 | 0 | 0 | 1 | 0.01 | 0 | -9 | 0 | 0 | 0 | 0 | 0 | 0 | 0 | 0 |
| No Control (no def) | 0 | 0 | 0 | 0 | 1 | 0.01 | 0 | -9 | 0 | 0 | 0 | 0 | 0 | 0 | 0 | 0 |
| **Physical Subscale** |  |  |  |  |  |  |  |  |  |  |  |  |  |  |  |  |
| Pain Severity | 1 | 0.02 | 0 | 6 | 0 | 0 | 0 | 0 | 0 | 0 | 0 | 0 | 0 | 0 | 0 | 0 |
| Activities | 0 | 0 | 0 | 0 | 0 | 0 | 0 | 0 | 0 | 0 | 0 | 0 | 0 | 0 | 0 | 0 |
| Get Around | 0 | 0 | 0 | 0 | 0 | 0 | 0 | 0 | 0 | 0 | 0 | 0 | 0 | 0 | 0 | 0 |
| **Bold**: Statistically significant violations | | | | | | | | | | | | | | | | |
| #VI: number of violations found | | | | | | | | | | | | | | | | |
| #VI/#AC: average number of violations for all active pairs of items compared | | | | | | | | | | | | | | | | |
| ZSig: Number of statistically significant violations | | | | | | | | | | | | | | | | |
| Crit: Critical Value for the Violations | | | | | | | | | | | | | | | | |
