# Supplementary material for: Scoring the EQ-HWB-S: can we do it without value sets? A non-parametric item response theory analysis
Source: Qual Life Res. 2024 Feb 21;33(5):1211–22. doi: 10.1007/s11136-024-03601-7 (PMC11045574; doi:10.1007/s11136-024-03601-7)
Supplement: Supplementary file 6 — Supplementary file6 (DOCX 15 kb) [file 11136_2024_3601_MOESM6_ESM.docx]

Appendix F: Supplementary Exploratory Factor Analysis Results

| **Appendix F1: EQ-HWB-S Exploratory Factor Analysis (Oblique (oblimin) Rotated)** | | | | | |
| --- | --- | --- | --- | --- | --- |
|  | **1-factor solution** | | **2-factor solution** | | |
| Item | Factor1 Loadings | Uniqueness | Factor1 Loadings | Factor2 Loadings | Uniqueness |
| Exhausted | **0.738** | 0.455 | **0.661** | 0.137 | 0.449 |
| Concentrating/thinking | **0.791** | 0.375 | **0.804** | 0.018 | 0.338 |
| Sad | **0.823** | 0.324 | **0.888** | -0.050 | 0.256 |
| Anxious | **0.814** | 0.337 | **0.891** | -0.066 | 0.263 |
| Lonely | **0.786** | 0.382 | **0.855** | -0.056 | 0.317 |
| No Control | **0.815** | 0.336 | **0.752** | 0.121 | 0.324 |
| Pain (severity) | **0.491** | 0.759 | 0.069 | **0.583** | 0.613 |
| Daily Activities | **0.651** | 0.576 | 0.025 | **0.861** | 0.234 |
| Get Around | **0.582** | 0.661 | -0.065 | **0.886** | 0.272 |
|  | Eigenvalue | Percent of variance* | Correlation between Factors |  |  |
| Factor 1 | 4.80 | 86.6% | 0.517 |  |  |
| Factor 2 | 1.14 | 20.6% |  |  |  |
| **Bold**: factor loading>0.4  *These results were obtained using the principal-factor method to analyze the correlation matrix, which can lead to negative eigenvalues and cumulative proportions >1 | | | | | |

| **Appendix F2: EQ-HWB-S Exploratory Factor Analysis: Standardized residuals of correlations** | | | | | | | | | | | |
| --- | --- | --- | --- | --- | --- | --- | --- | --- | --- | --- | --- |
|  |  | Exhausted | Concentrating/ thinking | Sad | Anxious | Lonely | No Control | Pain (severity) | Daily Activities | Get Around |  |
| **1-factor solution** | Exhausted | 0 | 1.961 | -0.382 | 0.710 | -1.081 | -0.765 | **3.440** | -0.374 | -1.656 | **2-factor solution** |
|  | Concentrating/ thinking | 2.678 | 0 | 0.359 | 0.815 | -0.772 | -0.199 | 0.163 | -0.319 | -0.011 |  |
|  | Sad | 0.559 | 2.757 | 0 | 0.635 | 1.304 | 0.425 | -0.429 | 0.250 | 0.026 |  |
|  | Anxious | 1.708 | **3.343** | **4.012** | 0 | 0.439 | 0.122 | -0.369 | 0.241 | -0.293 |  |
|  | Lonely | -0.149 | 1.595 | **4.538** | **3.804** | 0 | 1.748 | -0.916 | -0.010 | 0.537 |  |
|  | No Control | -0.372 | 0.801 | 1.771 | 1.530 | **3.108** | 0 | -1.059 | 0.478 | 0.753 |  |
|  | Pain (severity) | 1.881 | **-3.751** | **-5.683** | **-5.877** | **-6.110** | **-3.249** | 0 | 0.378 | 0.227 |  |
|  | Daily Activities | -2.608 | **-6.006** | **-7.416** | **-7.799** | **-7.621** | -2.740 | **12.579** | 0 | 2.930 |  |
|  | Get Around | **-4.069** | **-6.232** | **-8.340** | **-9.046** | **-7.797** | -2.769 | **13.370** | **22.919** | 0 |  |
|  | **Bold:** standardized residuals > \|3\| | | | | | | | | | |  |
|  | **Grey cells:** results from a 2-factor solution | | | | | | | | | |  |
|  | **White cells:** results from a 1-factor solution | | | | | | | | | |  |
